# Fatigue Crack Growth Behaviour of Precipitate-Strengthened CuNi_2_Si Alloy under Different Loading Modes

**DOI:** 10.3390/ma13102228

**Published:** 2020-05-12

**Authors:** Bing Yang, Yifan Li, Yahang Qin, Jiwang Zhang, Bo Feng, Zhen Liao, Shoune Xiao, Guangwu Yang, Tao Zhu

**Affiliations:** State Key Laboratory of Traction Power, Southwest Jiaotong University, Chengdu 610031, China; yb@swjtu.edu.cn (B.Y.); yihaifengfan000@163.com (Y.L.); zhangjiwang@swjtu.edu.cn (J.Z.); 13893277382@163.com (B.F.); liaozhen6@163.com (Z.L.); snxiao@swjtu.edu.cn (S.X.); gwyang@swjtu.edu.cn (G.Y.); zhutao034@swjtu.edu.cn (T.Z.)

**Keywords:** Cu–Ni–Si alloy, fatigue performance, loading mode, crack growth

## Abstract

In this study, fatigue crack tests of CuNi_2_Si alloys using the replica technique under symmetrical tensile-compression loading, and rotational-bending loading were carried out with the same nominal stress amplitude. Observation and analysis results indicate that under different load types, the cracks display a trend of slow initiation growth and then rapid growth. The critical point is identified at the approximate value of 0.8 of the fatigue life fraction, and the crack growth rate of the sample under tensile-compression load is approximately an order of magnitude higher than that under rotational-bending load, resulting in the average life of the former being significantly shorter than the latter. Combining the observation results of the fractographical analysis and the surface-etched sample replica film, it can be seen that whether it is a tensile-compression load or a rotational-bending load, cracks mainly propagate in intergranular mode after initiation.

## 1. Introduction

Cu–Ni–Si alloy is a precipitation-strengthened copper alloy with good mechanical properties, fatigue, corrosion resistance [1] and electrical conductivity [2]. It has been widely used for electrical components, such as electrical connectors, circuit breakers, relays and railway contact net positioning clamps, and rings [3,4]. However, there are still some challenges in the practical applications of CuNi_2_Si alloys. Considering positioning clamps and rings as an example, owing to the complex load such as the impact of the pantograph sweeping over the contact line and the cyclic load caused by wind load during service, there is a risk of fatigue fracture failure, threatening railway operation safety. Therefore, it is necessary to study the fatigue fracture performance of CuNi_2_Si alloy under various load types to provide a reference for related reliability and safety assessment.

Several studies have been carried out within the country as well as internationally on the fatigue fracture properties of Cu–Ni–Si alloys. Yang et al. [1] investigated the effects of specimens with and without cold working under both normal and salty atmospheric conditions on the fatigue properties and fracture behaviour of CuNi_2_Si alloys under bending rotation. Through the fatigue test and monotonic tensile tests of specimens under such conditions, it was found that the monotonic tensile strength and yield strength were evidently improved, whereas the elongation decreased by cold working. Under atmospheric conditions, the specimens with cold working displayed shear mode fractures whereas those without cold working displayed normal mode fractures.

Zhang et al. [5] studied the effect of micro-shot peening on the rotational-bending fatigue properties of CuNi_2_Si alloys under both normal and salty atmospheric conditions. It was found that the fatigue strengths of the micro-shot peening specimens at 10^7^ cycles were increased by 47% and 67% under normal and salty atmospheric conditions, respectively, compared to the un-peened specimens. The peened specimens under normal atmospheric conditions failed from the subsurface zone in the high-cycle fatigue region, whereas all the other specimens failed from the surface.

Gholami et al. [6,7] generated an ultrafine-grained microstructure in Cu–2.5Ni–0.5Si–0.06Mg (CuNi_3_Si_1_Mg) by applying a combination of microstructure refinement using swaging. The results indicated that compared to the precipitation-hardened non-swaged samples, the mechanical properties of the swaged samples after precipitation hardening are significantly improved. The fatigue strength of the ultrafine-grained CuNi_3_Si_1_Mg was approximately 1.6 times higher than the fatigue strength of the coarse-grained CuNi_3_Si_1_Mg. They further indicated that the initial microstructure affected the fatigue crack nucleation mechanism.

Atapek et al. [8] determined the S-N curve of the material by conducting fatigue tests on Cu-2.55Ni–0.55Si alloy under various stress levels. The fractured surfaces exhibited typical tracks indicating that the dimple morphology and intergranular or transgranular rupture are a function of the applied *σ*_max_, and that the crack propagation zone is narrow, and the final fracture zone is wide at high stress level.

Goto et al. conducted a series of studies on Cu–6Ni–1.5Si alloy [9,10,11] and found that the microstructure and treatment process have a certain effect on the fatigue performance of the alloy. The crack initiation life and propagation life of Cu–6Ni–1.5Si alloy with discontinuous/cellular precipitation are different, and the fracture morphology of the two is quite different [10]; the two processes of air cooling and water quenching have an effect on the fatigue strength and the fatigue strength of the air-cooled Cu–Ni–Si alloy was 1.1 times higher than that of the water-quenched alloy, while the tensile strength was only 75% that of the water quenched alloy [11].

Li et al. [12] investigated the fatigue fracture behaviour of coarse-grained copper under cyclic tensile-compression and torsion loadings and compared the cyclic stress response and dislocation mode of coarse-grained copper under two fatigue tests. They found that the fatigue crack initiation, propagation and fracture of the specimen depended on the loading mode; the initiation and early propagation of fatigue cracks were mainly controlled by the direction of the maximum shear stress.

These research results provide good theoretical support for the engineering application of Cu–Ni–Si alloys. In terms of load types, there are already researches on the impact of fatigue performance on tensile-torsional loads [13,14,15], bending-torsional loads [16,17] of steel and on bending-torsional loads of aluminium alloys [18,19,20]. Chaves et al. [21] conducted fatigue tests on 7075-T6 aluminium alloy under three conditions of tension, torsion and in-phase biaxial loading. The results show that the ratio between the pure torsion and tension endurance limits was 0.58, and for the three types of loads, the crack initiation point was close to the maximum principal stress point, and the crack direction was close to the maximum principal stress direction. Wu [22] studied the fatigue performance of LZ50 railway axle steel under two load types of rotational bending and axial tensile-compression. The results show that short fatigue cracks generally originated from ferrite grain boundaries or inside ferrite grains with lower hardness. The crack growth rate under tension-compression load is higher than that under rotational bending load, but both showed two typical decelerations. However, comparative studies on the fatigue crack behaviour of the copper alloy under various loading modes are still unavailable. In this study, fatigue crack replica tests of CuNi_2_Si alloy specimens with the same nominal stress level under two types of loading modes, namely, symmetrical tensile-compression and rotational-bending, are carried out to explore the effect of the type of load on the crack initiation and propagation behaviour.

## 2. Materials and Tests

### 2.1. Materials

The test materials were derived from CuNi_2_Si alloy round bar blanks. The chemical composition is presented in Table 1 and the mechanical performance data are presented in Table 2 [1]. Vickers hardness was measured using HVS-1000Z micro-hardness tester with a load of 0.25 N for 10 s.

**Figure 1:**
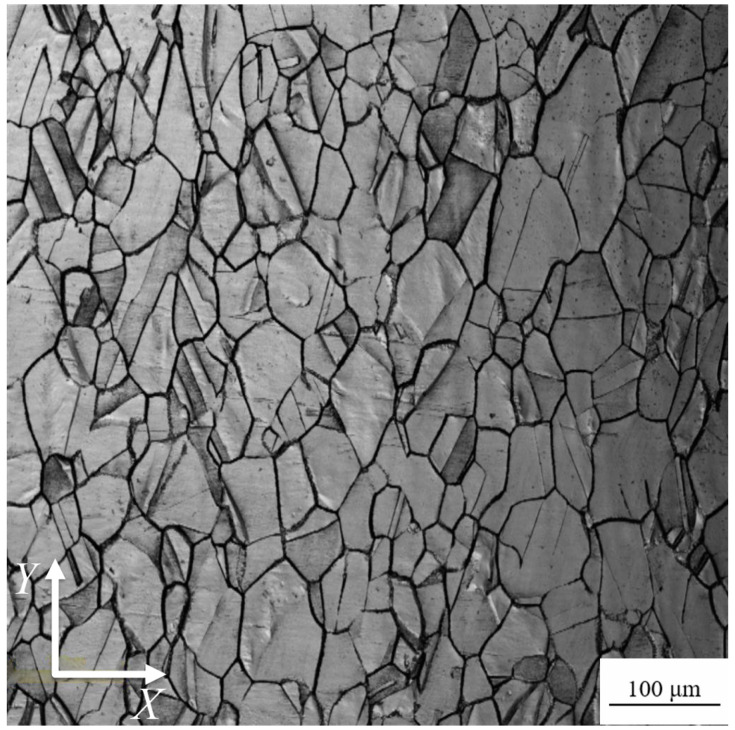
Metallurgical photograph of CuNi_2_Si alloy.

Figure 1 shows the metallurgical structure of the material after heat treatment at 400 °C for 2 h, where the *Y*-direction is consistent with the axial direction of the round bar blank.

The material is composed of α-Cu with lots of twins, and the grains mainly extend in the *Y*-direction. Observations and statistics using the Olympus OLS4100 confocal laser scanning microscope by means of the line method indicated that the average grain size of the materials in the *X*- and *Y*-directions were 51.63 and 85.31 μm, respectively.

### 2.2. Fatigue Tests

**Figure 2:**
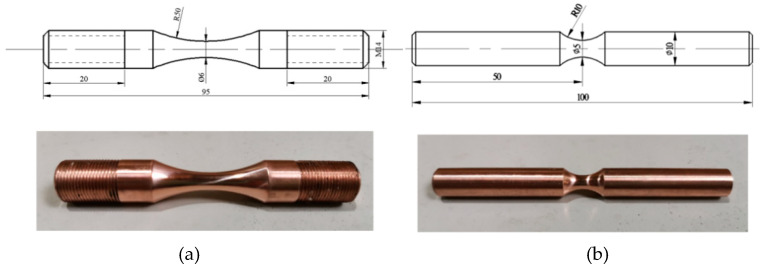
Specimens and dimensions in millimetres: (**a**) TC specimen; (**b**) RB specimen.

The axial tensile-compression (TC) and rotational-bending (RB) specimens depicted in Figure 2 were processed separately. 

To facilitate the copying, the surface of the arc segment of the sample was polished by emery papers with a grit of size 800–2000 before the test. Thereafter, the Al_2_O_3_ water-soluble suspension with particle sizes of 1 and 0.5 μm was used to polish the surface of the sample until a mirror effect was obtained.

**Figure 3:**
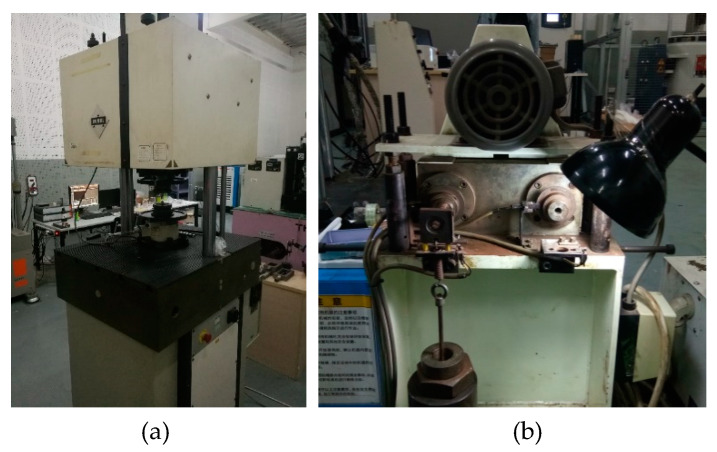
Test equipment: (**a**) Rumul; (**b**) Horks.

Two types of tests were performed using the Rumul 250 kN high-frequency fatigue test machine manufactured by Russenberger Prüfmaschinen AG in Neuhausen, Switzerland and Horks RB4-3150 rotational-bending fatigue test machine manufactured by HORKOS CORP in Hiroshima, Japan (Figure 3) with a nominal stress level of 240 MPa. The stress ratio of both the TC tests and the RB tests is *R* = −1, and the loading frequencies are 80 Hz and 52.5 Hz, respectively. 

To obtain surface crack initiation and propagation information during the test, the procedure was stopped at a predetermined number of load cycles, the cellulose acetate membrane was softened with methyl acetate and it was pasted on the smallest cross section of the specimen. For subsequent observations, it was flattened with a glass slide until the replica film was completely dry. Existing studies have reported that the replica method can effectively reproduce the initiation and propagation of fatigue cracks, and that the technology has no significant effect on the fatigue life of materials [23,24,25].

## 3. Results and Discussion

### 3.1. Replica Film Observation

A total of six TC and RB specimens each were obtained—five smooth surface specimens and one etched surface specimen each. The smooth surfaces of the TC and RB specimens were numbered TC1 to TC5 and RB1 to RB5, respectively.

**Figure 4:**
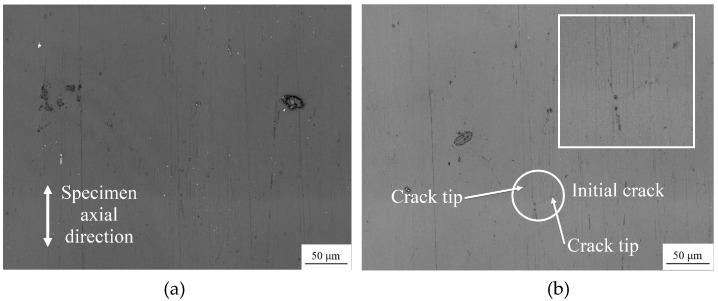

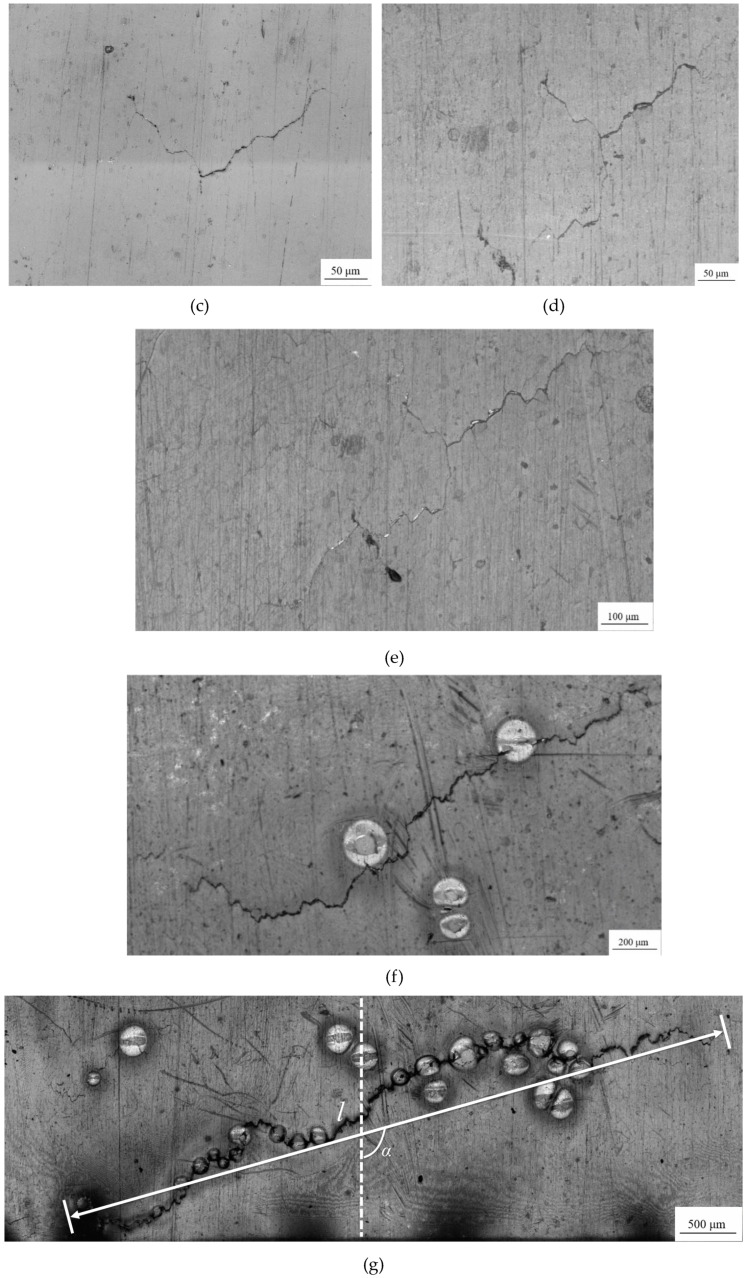
Replica photos for specimen TC2 under different number of cycles: (**a**) *N* = 0 cycles; (**b**) *N* = 200 cycles; (**c**) *N* = 4000 cycles; (**d**) N = 14,000 cycles; (**e**) *N* = 30,000 cycles; (**f**) *N* = 53,000 cycles; (**g**) *N* = 59,000 cycles.

Taking the sample TC2 under symmetrical tensile and compression load as an example, Figure 4 presents the photographs of the replica film during the whole process of crack initiation and propagation on the surface of the sample. The fatigue life of this sample was *N_f_* = 59,900 cycles.

It can be seen in Figure 4a that when *N* = 0, the surface of the sample is intact and there are no evident cracks but slight scratches caused by processing remain. After 200 load cycles (Figure 4b), crack initiation is visible. The angle between the line connecting the crack tips and the axial direction of the specimen is defined as the crack angle *α*, and the projection length *l* of the connecting line perpendicular to the axial direction of the specimen is the crack length *a*, i.e., *a* = *l* × sin*α*. At this time, the crack length was approximately 21.8 μm, which is smaller than the average grain size, and did not initiate at the scratch caused by processing.

As the cycle numbers increased, the cracks continued to propagate (Figure 4c), with new cracks (Figure 4d) and mergers between cracks (Figure 4e), but until *N* = 30,000 cycles, the cracks were still in a relatively slow propagation state, with a length of approximately 819.1 μm. Subsequently, the crack growth rate increased rapidly, from *N* = 53,000 cycles (Figure 4f) to *N* = 59,000 cycles (Figure 4g), with the crack length increasing rapidly from approximately 2162.8 to 6659.8 μm, and the specimen failed after another 900 cycles.

Observing other specimens of the tensile-compression and rotational-bending tests, it was found that there were both single and multiple cracks under the stress level of 240 MPa.

### 3.2. Fracture Analysis

**Figure 5:**
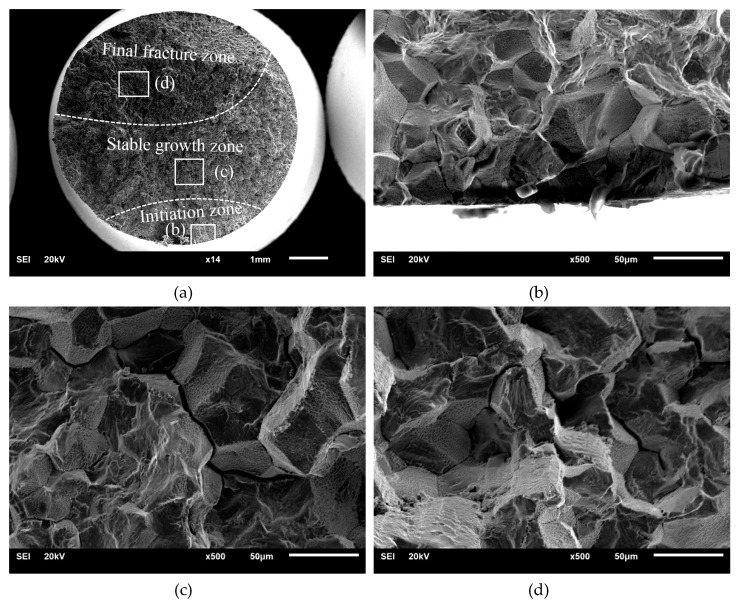
Fracture surface observations of tensile-compression specimen: (**a**) Fracture surface observation; (**b**) Fatigue crack initiation zone; (**c**) Fatigue crack growth zone; (**d**) Final fracture zone.

**Figure 6:**
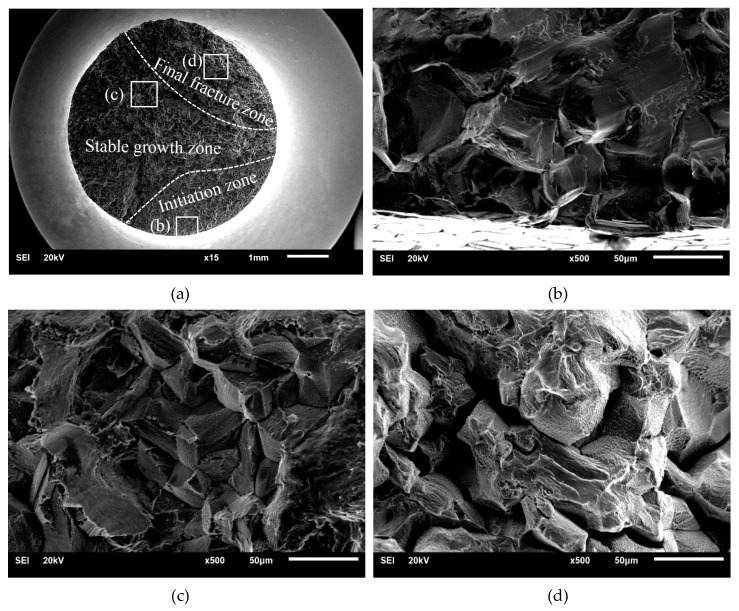
Fracture surface observations of rotational-bending specimen: (**a**) Fracture surface observation; (**b**) Fatigue crack initiation zone; (**c**) Fatigue crack growth zone; (**d**) Final fracture zone.

The fractures of the tensile-compression specimen and rotational-bending specimen are depicted in Figure 5 and Figure 6. The fatigue fractures of the rotational-bending specimens and tensile-compression specimens are composed of three stages: crack initiation, stable growth and final fracture zones. All the cracks originated from the crystal slip on the surface of the specimen. In the crack propagation zone, several intergranular facets can be observed, which indicates that the cracks mainly propagate along the crystal boundaries. In Figure 5d and Figure 6d, tiny dimples are visible in addition to the intergranular facets in the transient fracture zone.

### 3.3. Crack Propagation Behaviour

**Figure 7:**
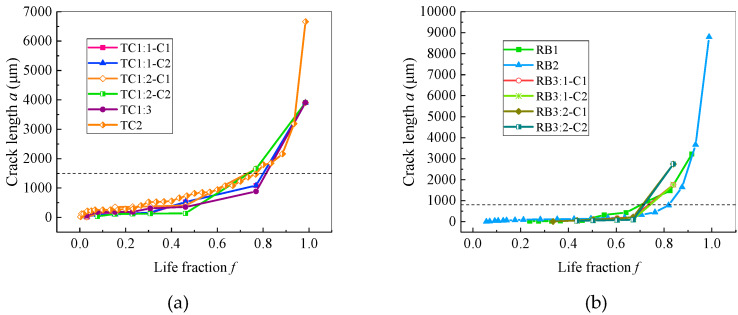
Curve of crack length a as a function of life fraction *f*: (**a**) TC specimens; (**b**) RB specimens.

Based on the measured crack lengths of the replica films, the curves of the crack lengths of the typical tensile-compression specimens TC1 and TC2 and the typical rotational-bending specimens RB1 to RB3 were drawn as a function of the fatigue life fraction (cycle times *N*/fatigue life *N_f_*) as depicted in Figure 7. Multiple crack initiations and mergers may exist in the crack initiation and propagation of a single specimen, therefore to distinguish among various crack sources and cracks, the selected symbol “*i*-C*j*” indicates the cracks originated at the *j*th crack source in the *i*th crack; e.g., “1-C2” indicates the cracks that originated at the second crack source in the first crack.

It can be seen from Figure 7 that the crack length for both the rotational-bending specimen and the tensile-compression specimen exhibit a behaviour of slowly increasing first and then rapidly expanding, and the life fraction of 0.8 is the critical point between the two. The difference is that the tensile-compression specimens grow faster than the rotational-bending specimens during the slow crack growth stage. In the present study, the crack size corresponding to the turning point between slow and fast crack growth (with a life fraction of 0.8) is defined as the critical crack length. It can be seen from Figure 7 that the critical crack length of the rotational-bending specimen is approximately 800 μm, and that of the tensile-compression specimen is approximately 1500 μm. The critical crack length of the TC specimen is much larger than that of the RB specimen, mainly because the crack growth rate of the TC specimen is much higher than that of the RB specimen during the slow propagation stage, which results in longer crack of the TC specimen than that of the RB specimen before the life fraction reaches 0.8.

**Figure 8:**
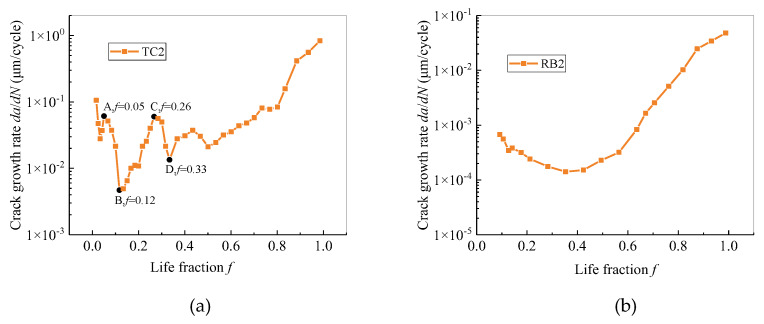
Curve of crack growth rate as a function of life fraction: (**a**) TC specimen; (**b**) RB specimen.

**Figure 9:**
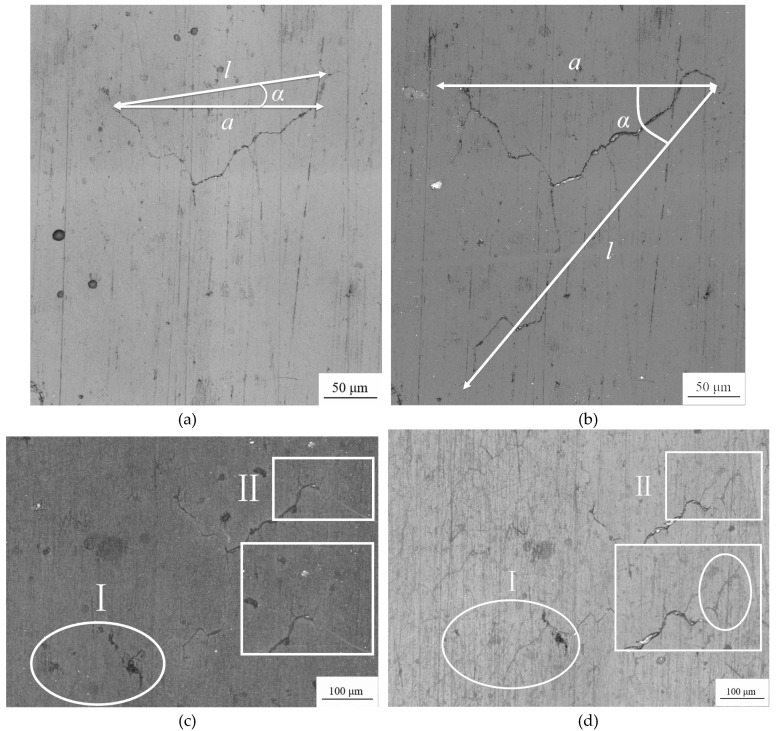
Cracks corresponding to decreases in crack growth rate of specimen TC2: (**a**) *N* = 3000 (*f* = 0.05); (**b**) *N* = 7000 (*f* = 0.12); (**c**) *N* = 16,000 (*f* = 0.26); (**d**) *N* = 20,000 (*f* = 0.33).

One representative sample is selected from each of the rotational-bending and tensile-compression samples, and the curves of the crack growth rate as functions of the life fraction and crack length are plotted as presented in Figure 8 and Figure 9, respectively. The crack growth rate is calculated using the five-point method [26].

It can be seen from Figure 8 that under the two types of loads, the crack growth rate is relatively low in the early stage; when the life fraction exceeds 0.8, the growth rate increases rapidly. The tensile-compression specimens displayed rapid expansion in the initial stage of crack initiation, and the growth rate demonstrated a fluctuating phenomenon in the early stage of crack propagation. Comparing the growth rates of the specimens under the two types of loads, it can be found that the propagation rate of the tensile-compression specimens is an order of magnitude greater than that of the rotational-bending specimens. 

In addition, from the curve of crack growth rate of TC2, it can be found that the crack growth rate clearly exhibits two decelerations, which started from point A (*f* = 0.05) and point C (*f* = 0.26) to point B (*f* = 0.12) and point D (*f* = 0.33), respectively. Combining the photographs of the replica film at these moments, the reason for the decrease in crack growth rate could be as follows:

When the crack growth rate of TC2 first decreased at point A (*f* = 0.05), as shown in Figure 9a,b, the crack did not propagate along any crack tip, but initiated from the middle of the crack in the other direction. The change of the calculated projected crack length was very slight, resulting in a minimum value of crack propagation rate at this moment. When the crack growth rate decreased for the second time at point C (*f* = 0.26), as shown in Figure 9c,d, the cracks had grown in the area I, corresponding to the initial stage from point C to point D, where the crack growth rate was basically a constant value. However, in area II, a small crack initiated but was not merged with the main crack. The initiation of the small crack did not cause the length change of the main crack. At this moment, the crack growth rate began to decrease. As the test went on, the newly-initiated crack was merged with the main crack, and then the propagation of the crack came back to normal.

**Figure 10:**
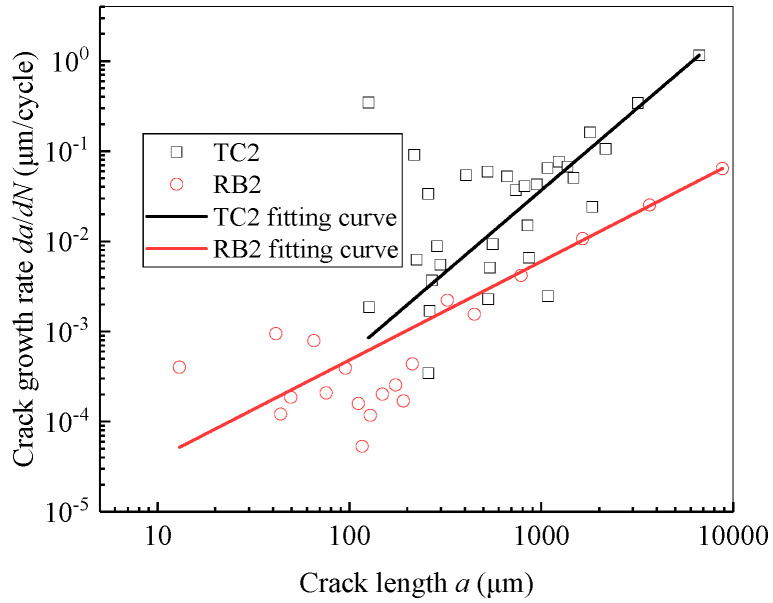
Fitting curve of crack growth rate as function of crack length.

For a more intuitive comparison, *da*/*dN*–*a* curves of the typical samples TC2 and RB2 are constructed using a formula similar to the Paris formula [27] and plotted in Figure 10. The results curve-fitting are as follows:

TC2: *da*/*dN* = 1.3 × 10^−7^*a*^1.8^ (1)

RB2: *da*/*dN* = 3.2 × 10^−6^*a*^1.1^ (2)

The squared-fit coefficients of TC2 and RB2 were 0.89 and 0.99, respectively. It is easy to see that during the crack growth process, although the crack growth rate fluctuates, the overall trend is that of an increase and the propagation rates of the tensile-compression specimens are significantly larger than those of the rotational-bending specimens. It can be seen from reference [5] that the crack growth rate of CuNi_2_Si alloy obtained by carrying out the rotational bending test basically shows the same order of magnitude as that in the present study.

**Figure 11:**
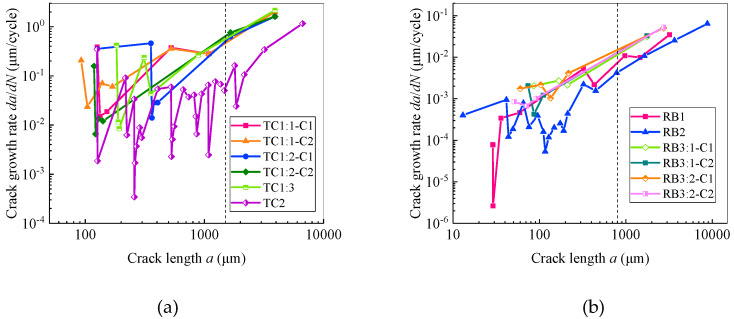
Curve of crack growth rate as function of crack length: (**a**) TC specimens; (**b**) RB specimens.

In the double-logarithmic coordinate system depicted in Figure 11, it can be seen that irrespective of whether the specimen is rotational-bending or tensile-compression, when the crack length is below the respective critical crack size (obtained from Figure 7), the crack growth rate does not increase with the increase in crack length. In contrast, both have large fluctuations. However, when the crack size is larger than the critical value, the crack growth rate almost increases with increase in the crack length. Similar phenomena were observed by Connolley et al. [28] on the Inconel 718 alloy, Zhu et al. [25] on nickel-based alloys and Deng et al. [29] on the nickel-based alloy GH4169.

**Figure 12:**
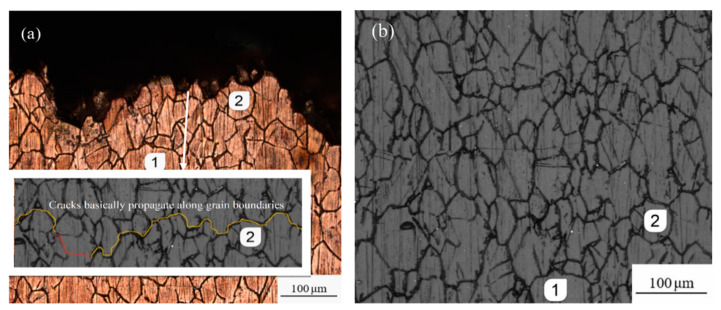
Side view of fracture and corresponding replica film (etched TC specimen): (**a**) the fracture side view of the etched surface specimen; (**b**) the photograph of the replica film before testing.

**Figure 13:**
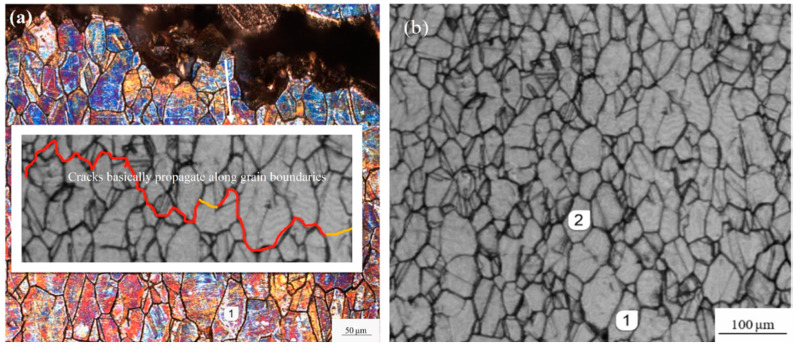
Side view of fracture and corresponding replica film (etched RB specimen): (**a**) the fracture side view of the etched surface specimen; (**b**) the photograph of the replica film before testing.

To further determine the fracture mode of the specimens, Figure 12 and Figure 13 present the fracture side view of the etched surface specimens under tensile-compression and rotational-bending loading, respectively (Figure 12a and Figure 13a), and the corresponding photographs of the replica films before testing (Figure 12b and Figure 13b). Comparing the fractures and the replica films, it can be seen that, basically, the surface crack overlaps the grain boundary irrespective of which loading mode is applied. Again, this indicates that the cracks mainly propagate in an intergranular fracture mode.

### 3.4. Discussion

Comparing the crack propagation information obtained on TC and RB specimens (Figure 7, Figure 8, Figure 9 and Figure 10), it can be concluded that the crack growth of the CuNi_2_Si alloy surface is faster under tensile and compression load cycles, and that the crack growth rate of tensile and compression specimens is an order of magnitude greater than that of rotational-bending specimens. This leads to the fatigue life of tensile and compression specimens being significantly lower than that of rotational-bending specimens. The average life of tensile and compression specimens in the test is 30,000 cycles, whereas that of rotational-bending specimens is 750,000 cycles.

Regarding the impact of the load types of tension-compression and rotational bending on fatigue life, studies have shown that under the same nominal stress, the fatigue life of the tensile-compression test is shorter than that of the rotational bending test [30,31]. The differences can be explained by the existence of stress gradients [31]. That is to say, under the same nominal stress, there is no stress gradient in the cross section of the tensile-compression specimen but a significant one in that of the rotational bending specimen, which leads to that the fatigue life of tensile-compression specimen is relatively shorter.

Based on the fracture observation of tensile-compression specimens and rotational-bending specimens, it can be concluded that the cracks initiate because of crystal slip on the surface of the specimens. Under fatigue loading, fatigue cracks nucleate at twins or high-angle grains and dislocations accumulate at the grain boundaries; thus, fatigue cracks propagate along the grains. 

The grains of the copper alloy can be observed in both the crack propagation zone and the transient fracture zone; there is also separation between adjacent grains in the transient fracture zone. Combined with the fracture side views and the replica film photographs of the corrosion specimens, it was confirmed that both the tensile-compression and rotational-bending specimens demonstrate an intergranular fracture mode.

## 4. Conclusions

In this study, through the short crack replica test of CuNi_2_Si alloy under tensile-compression and rotational-bending loading, the initiation and propagation of the short cracks and failure modes of the specimens were compared and analysed and the following conclusions were reached:

1. Under the two types of loading, fatigue cracks exhibit slow growth and rapid expansion. Before the point at which the life fraction is equal to 0.8, the crack length and crack growth rate are at low levels. However, the expansion rate of tensile and compression specimens is an order of magnitude greater than that of rotational-bending specimens. In this study, the average life of tensile and compression specimens is 30,000 cycles, which is significantly lower than the 750,000 cycles of rotational-bending specimens.
2. For the tests carried out in this study, the critical crack sizes of the tensile-compression and rotational-bending samples are 1500 and 800 μm, respectively. When the crack length is less than the critical crack size, the crack growth rate of the two does not increase with the increase in the crack length. In contrast, both samples display a large fluctuation, but when the crack size is larger than the critical crack size, the crack growth rate increases almost as the crack length increases.
3. For both the TC and RB specimens, the cracks basically propagate along the boundaries and indicate an intergranular fracture mode.

## Figures and Tables

**Table 1:** Chemical composition of CuNi_2_Si alloy (wt %).

Cu	Fe	Mn	Ni	Pb	Si	Sn	P	Al	Zn
97.5	0.1395	<0.001	1.75	0.001	0.482	0.033	0.012	0.002	0.0261

**Table 2:** Mechanical properties of CuNi_2_Si alloy.

Ultimate Tensile Strength (MPa)	Yield Strength (MPa)	Elongation (%)	Vickers Hardness
646	583	14.0	202
